# Associations between music education, intelligence, and spelling ability in elementary school

**DOI:** 10.2478/v10053-008-0082-4

**Published:** 2011-03-04

**Authors:** Katrin Hille, Kilian Gust, Urlich Bitz, Thomas Kammer

**Affiliations:** 1 Transfercenter for Neuroscience and Learning, University of Ulm, Germany; 2 Department of Psychiatry, University of Ulm, Germany

**Keywords:** music education, intelligence, literacy, spelling, cognitive development

## Abstract

Musical education has a beneficial effect on higher cognitive functions, but questions arise whether associations between music lessons and cognitive abilities are specific to a domain or general. We tested 194 boys in Grade 3 by measuring reading and spelling performance, non verbal intelligence and asked parents about musical activities since preschool. Questionnaire data showed that 53% of the boys had learned to play a musical instrument. Intelligence was higher for boys playing an instrument (*p* < .001). To control for unspecific effects we excluded families without instruments. The effect on intelligence remained (*p* < .05). Furthermore, boys playing an instrument showed better performance in spelling compared to the boys who were not playing, despite family members with instruments (*p* < .01). This effect was observed independently of IQ. Our findings suggest an association between music education and general cognitive ability as well as a specific language link.

## Introduction

Active music performance relies on a demanding action-perception-loop calling for long periods of focused attention on dynamic visual, auditory, and motor signals. Given this extra training of high-level cognitive skills in children who learn to play an instrument, it can be asked whether making music enhances children’s performance in domains other than music.

 Positive relationships between playing an instrument and general cognitive abilities have been observed previously. In a retrospective design with 6- to 11-year-old children, Schellenberg (2006) found a correlation between the duration of music lessons and performance in an verbal and non-verbal IQ test as well as school performance. The effects on IQ and on academic performance were still observable in undergraduates that had been trained to play an instrument in childhood. Forgeard, Winner, Norton, and Schlaug (2008) observed a relationship between playing an instrument and higher cognitive functions in a sample of forty-one 8- to 11-year-old children who had at least 3 years of musical instruction. Beside motor learning and enhanced melodic discrimination, the authors also found enhanced vocabulary and nonverbal reasoning scores. 

However, no differences were found in a prospective study investigating 6- year old children between a group of 16 control children and 15 children who had weekly private keyboard lessons for 15 months (Hyde et al., 2009). Nevertheless, the authors were able to show near-transfer effects (motor and auditory skills) as well as structural brain changes for the keyboard group.

 In an experimental design, Schellenberg (2004) reported an effect on IQ using Wechsler’s WISC-III in 6-year-olds after keyboard or singing lessons for 36 weeks. The music group (+ 7.0 points) showed a larger increase than the control group taking drama lessons in the same time or waiting for piano lessons (+ 4.3 points). This finding contrasts the meta-analysis of Hetland (2000, Analysis 2) including five experimental studies about the effect of musical training on Raven’s IQ. 

Besides this broad effect of music on general cognitive performance, some studies also found associations with mathematical (Cheek & Smith, 1999; Vaughn, 2000) and spatial abilities (Hetland, 2000; Analysis 1).

Moreover, there seems to be a link between musical training and language abilities since musical training in childhood influences the development of auditory processing in the cortex (Fujioka, Ross, Kakigi, Pantev, & Trainor, 2006; Moreno & Besson, 2006). There is evidence that musical training is linked to language related aspects such as pitch processing (Moreno et al., 2009; Schön, Magne, & Besson, 2004; Wong, Skoe, Russo, Dees, & Kraus, 2007), speech prosody (Thompson, Schellenberg, & Husain, 2004), verbal memory (Chan, Ho, & Cheung, 1998; Ho, Cheung, & Chan, 2003; Jakobson, Cuddy, & Kilgour, 2003; Kilgour, Jakobson, & Cuddy, 2000). Additionally, musical aptitude was found to correlate with second language acquisition (Slevc & Miyake, 2006). Furthermore, associations of musical training and reading performance have been demonstrated in a normal population (Barwick, Valentine, West, & Wilding, 1989; Butzlaff, 2000; Lamb & Gregory, 1993) as well as in dyslexics (Overy, 2003).

The putative link between musical and language abilities is seen in the discrimination of rapid auditory events (Jakobson et al., 2003; Tallal & Gaab, 2006). Musical instrument training should improve auditory information processing, which in turn is crucial for the acquisition of reading and writing skills.

It is no longer the question whether or not musical training is associated with higher cognitive abilities, because there is growing evidence that it is. An unresolved issue however, is the nature and specificity of the link (Schellenberg & Peretz, 2008). It has been proposed that all specific relations observed so far can be explained by a carry-over effect of the relation between musical training and general abilities as measured by IQ (Schellenberg & Peretz, 2008). Indeed, such a dependency was always found in Schellenberg’s studies. Most of the previous studies showing a relation between musical training and specific abilities, such as language performance, did not measure general abilities. Therefore these studies could not report on the dependency of both.

Our correlational study addresses this unresolved issue of link-specificity by looking at a general association as well as at a specific language association of musical training.

## Methods

### Participants

We recruited 272 elementary school boys of Grade 3 aged 8 to 9 years from 26 schools in a southern German school district. The recruitment served two purposes. On the one hand, the boys were screened for an electroencephalographic study on auditory processing in normal and dyslexic children (Gust, 2009). Therefore we included only healthy boys who were native German speakers and had not repeated a class. On the other hand all screening data was used in combination with an additional parents’ questionnaire to answer the research question presented here.

The study followed the principles of the declaration of Helsinki and was approved by the local internal review board of the Medical Faculty, University of Ulm.

### Tests and questionnaire

We tested non-verbal intelligence with the German adaptation of Cattells Cultural Fair Intelligence Test - Scale 1 (CFT-1; Cattell, Weiß, & Osterland, 1997). The CFT-1 consists of five subtests (substitutions, labyrinths, classification, similarities, and matrices) and takes about 45 min to complete. This non-verbal IQ test was chosen to measure intelligence independently from progress in reading and writing.

Reading and spelling performance was tested with the Salzburger Lese- und Rechtschreibtest (SLRT; Landerl, Wimmer, & Moser, 1997). The SLRT is an individually given test assessing reading accuracy and reading speed for three word and two non-word reading subtests as well as spelling performance with regard to different types of spelling errors.

Parents filled out a questionnaire about the musical experience of their child during preschool and school years, including singing, listening to music, and playing an instrument, either at home or in an institutional setting such as children choir and music school. Additionally, we asked questions about the parental encouragement concerning non-musical activities. It was rated on a scale from 1 (*never*) to 7 (*more than once daily*) how often adults engaged with the boys in activities like looking together at picture books, reading books to the boys, telling stories to the boys, encouraging boys to draw and paint, or being at the playground with them. A composite score of “parental investment” was calculated from these ratings.

Lastly, parents were asked if any family member is playing an instrument. We expect that boys who play an instrument differ from boys that do not play an instrument. The existence of family members who play instruments allows to control for any unspecific differences, such as the family value of playing an instrument, or the minimum family income to allow for financing an instrument and lessons.

Descriptive and inferential statistics were computed using STATISTICA 7.1 (StatSoft, Inc. Tusla, OK, USA).

## Results

Two hundred and six parents completely answered and sent back the questionnaire on the musical experience of their boys (76% return rate). Table 1 summarizes the overall musical experience of the boys. One quarter had experience in singing in a choir, and half of the boys learned playing an instrument or did so in the past. Table 2 provides a breakdown of the boys who learned an instrument according to the age at which boys started musical instrument training.

**Table 1.** Overall Musical Experience of Boys.

	No	Yes
Choir	153 (74,3)	53 (25,7)
Course „First Experiences With Music”	142 (68,9)	64 (31,1)
Playing an instrument	97 (47,1)	109 (59,2)

*Note*. In the course „First Experiences With Music” the boys were trained to listen, to sing and dance together, and to play on instruments such as glockenspiel and woodblock.

A complete data set on non-verbal IQ, spelling and reading with the SLRT resp. was available for 194 of the 206 boys whose parents returned the questionnaire on musical experience.

**Table 2.** Start of Playing an Instrument.

Age	6 or younger	7	8	9
How old was the boy when he star-ted to play an instrument? *n* (%)	33 (30,6)	36 (33,3)	26 (24,1)	13 (12,0)

*Note*. Instrument types were recorder (*n* = 56), piano or keyboard (*n* = 33), guitar (*n* = 11), drum set, drum, trumpet, French horn, saxophone, accordion, melodica, baritone horn, violoncello, glockenspiel, xylophone.

### Playing a musical instrument and intelligence

**Figure 1.**
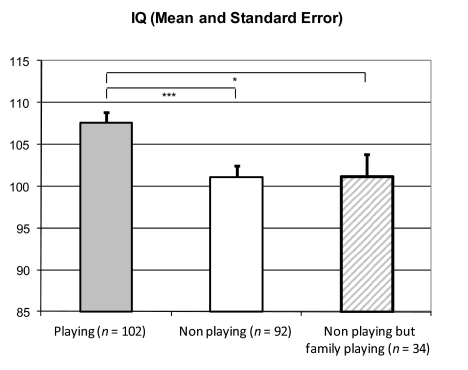
Mean differences of non-verbal IQ in relation to playing an instrument. *p < .05. ***p < .001.

In our sample intelligence showed a normal distribution with a mean of 104.5, a standard deviation of 13.6, a minimum of 72 and a maximum of 142. The non-verbal IQ was higher for boys playing an instrument, *t*(192) = 3.45, *p* <. 001 (see Figure 1). The size of the effect (*d* = 0.50) was at a medium level (Cohen, 1988). To control for differences in family values and family income boys who lived in families without musical instruments (*n* = 58) were excluded. The effect on intelligence remained, *t*(134) = 2.40, *p* < .02, *d* = 0.46 (Figure 1), when we compared the boys not playing (from families with members playing musical instruments) with the boys playing an instrument themselves.

**Figure 2.**
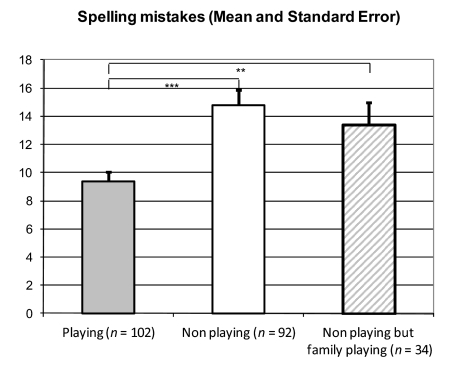
Mean differences for spelling mistakes in relation to playing an instrument. **p < .01. ***p < .001.

No difference in non-verbal IQ was found between boys who have sung in a choir and those who did not, *t*(192) = 1.53, *p* = .127. For boys who took part in a course on “First Experiences With Music” a higher non-verbal IQ was found, *t*(192) = 2.76, *p* < .01, *d* = 0.41. However, when families without musical instruments were excluded, this difference disappeared, *t*(134) = 1.75, *p* = .083. “Parental investment” correlated weakly with non-verbal IQ, *n* = 183, *r* =.156, *p* < .05.

### Playing a musical instrument and performance in reading and spelling

Spelling performance was better for boys playing an instrument as measured by the spelling mistakes made in the SLRT, *t*(192) = 4.22, *p* < .0001, *d* = 0.60. This effect remained after excluding the families without instruments, *t*(134) = 2.78, *p* < .01, *d* = 0.51.

A weak correlation between spelling mistakes and non-verbal IQ (*r* = -.17, *p* < .05) was found in our sample: The more intelligent the students the fewer spelling mistakes they made. To eliminate the effect of non-verbal IQ an ANCOVA was performed that confirmed the relationship between playing an instrument and spelling independently of non-verbal IQ, *F*(1, 191) = 13.96, *p* < .001; also after families without instruments were excluded, *F*(1, 133) = 5.36, *p* < .05.

Reading performance was accessed by reading speed and by reading mistakes as measured by the SLRT. Only for the reading time the boys who play an instrument showed an advantage, *t*(192) = 2.02, *p* < .05, *d* = 0.29; but this better performance disappeared when families without musical instruments were excluded, *t*(134) = 0.53, *p* = .60, *d* = 0.09.

The other variables (singing in a choir, taking part in a course on “First Experiences With Music”, “Parental Investment”) were not associated with reading or spelling performance.

### Playing a musical instrument and performance in reading and spelling in low performers

The results so far described were obtained from the whole group of boys. In the following analysis we focus on low-performer in terms of spelling. Low performers were defined as the quarter of boys (*n* = 51) with the highest number of SLRT spelling mistakes. Not all of these low-performers showed a spelling deficit as defined by the ICD-10. Only 17 of them exhibited a spelling performance on or below 10% of the population accompanied by an IQ within the normal range.

Boys who played an instrument were underrepresented in the lowest quartile of spelling performance: Only 27.5% of boys in the lowest quartile played an instrument whereas 61.5% of boys of the better quartiles were active musicians. Comparison between lowest quartile and all other quartiles combined proved a significant difference, X^2^(1) = 17.52, *p* < .0001; that turned into tendency towards significance after families without instruments were excluded, X^2^(1) = 6.86, *p* = .076.

## Discussion

Boys from a non-selected sample of third grade elementary school who play an instrument have shown a higher non-verbal IQ and were better in a formal spelling test compared to boys who did not play an instrument. The effects remained when controlled for musical interests by excluding families without instruments. The positive effect on reading vanished after this exclusion. The effect on spelling was independent of the influence from non-verbal IQ. A closer look at the distribution in spelling performance showed that only students in the lowest quartile differ from the others with respect to playing an instrument.

Our sample is not representative for the whole school population because of our inclusion criteria: male sex, right handedness, native German speakers, no class repetition. For the purpose of the current study the exclusion of girls seems to be a disadvantage. Advantageous, however, is the exclusion of children without native German language as our study needs a homogeneous group in terms of language acquisition. We did not focus on students suffering from dyslexia but included the whole range reading and spelling performance in a normal school population. The obtained return rate of the parents’ questionnaire about the musical experience of their child (76%) is within acceptable limits.

A further restriction of our study is the retrospective design. The results do not clarify a causal relation from music education to cognitive performance, they only demonstrate correlations. Any questionnaire covering the past might introduce a positive bias. But this should not be a problem for the core of our results, because we do not expect that parents give a wrong answer to the simple question “Does your child play an instrument?” We did not specify this response: We included children who received recorder group lessons for 30 min per week at age 8 for some weeks as well as children who played instruments with up to 4 hr individual lessons weekly starting at the age of 5. Given that weak inclusion criterion our results may even underestimate the observed effect.

### Relation to general cognitive abilities

 A positive effect of playing an instrument on general cognitive abilities has been observed previously. Schellenberg (2004) reported an effect on IQ using Wechsler’s WISC-III in 6-year-olds after keyboard or singing lessons for 36 weeks. In our study, the differences between the two groups were larger regarding both the IQ difference (Schellenberg delta 2.7 points vs. delta 6.6 points) and the effect size (Schellenberg d 0.35 vs. d 0.52). However, the studies differed considerably. For instance, we did not measure changes over time but compared two groups classified by the parents’ questionnaire. Furthermore, we also focused on reading and writing performance and have therefore chosen a non-verbal IQ test to measure intelligence independently from progress in reading and writing. Schellenberg (2004) did not observe a difference between verbal and non-verbal subtests in his investigation. 

### Relation to specific cognitive abilities for reading and writing

The putative link between musical and language abilities is seen in the discrimination of rapid auditory events (Jakobson et al., 2003; Tallal & Gaab, 2006). Our results indicate a stronger link of playing an instrument in respect to spelling as to reading performance. This result is in contrast to findings reported in a meta-analysis (Butzlaff, 2000) and has, to the best of our knowledge, not been reported before.

Our findings can be explained in different ways. Firstly, it has to be taken into consideration that the German language has better phoneme–grapheme mapping than the English language. Therefore, it might be feasible that native German speakers benefit from auditory training such as playing an instrument in regard to the discrimination of rapid auditory events which in turn helps them to decipher the spelling of the German words.

Another explanation can be found in the double deficit hypothesis of dyslexia (Wolf & Bowers, 1999). According to this hypothesis, spelling deficits are associated with a phonological deficit whereas dysfluent reading is associated with a naming speed deficit (Wimmer & Mayringer, 2002; Wimmer, Mayringer, & Landerl, 2000). The naming speed deficit is supposedly not due to auditory information processing but rather to lexical access. In contrast, musical training mainly affects sound processing and therefore spelling capability.

**Figure 3.**
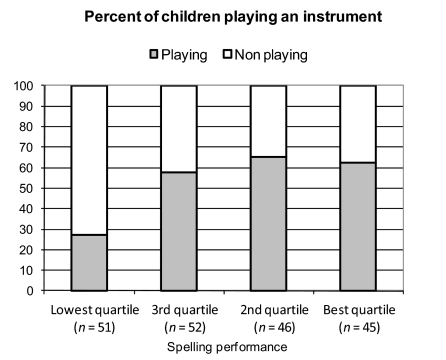
Percent of boys playing an instrument in relation to spelling performance (whole sample).

In line with these results, studies with dyslexic risk populations of 6-year old children and a dyslexic population of 9-year old children demonstrated a positive effect of musical lessons on spelling performance and phonological abilities but not on reading (Overy, 2003). It therefore appears from these studies that children with particularly low reading and spelling abilities benefit most from playing a musical instrument. We cannot exclude that low performers dislike playing an instrument and therefore cause the observed group differences. Our data (Figure 3) show a leap between the percentages of players in the two lowest quartiles (delta 30%) that cannot be seen between the other quartiles (delta max. 8%). This pattern suggests a kind of threshold.

**Figure 4.**
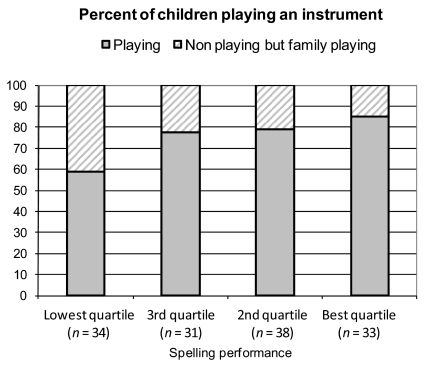
Percent of boys playing an instrument in relation to spelling performance (sub sample of families with musical instruments).

In our sample, the active participation in a choir or the lessons “First Experiences With Music” did not show the benefits found when children were playing musical instruments. It cannot be ruled out that this negative finding is based on intensity or quality of the musical activities. Another explanation could be the differences in specific motor skill between singing and playing an instrument. Further studies that control for intensity and quality of the musical courses might clarify if there is a specific advantage in playing an instrument.

### Specific versus general effects

It has been proposed that all specific relations observed so far can be explained by a carry-over effect of the relation between musical training and general abilities as measured by IQ (Schellenberg & Peretz, 2008). Indeed, in Schellenberg’s studies such dependency was observed. Our data contrast these observations. We observed both a relation to general abilities as measured by non-verbal IQ and a relation to spelling performance that was still observed when controlled for general abilities. The relation between musical training and spelling performance may alternatively be explained by an increase of crystallized intelligence. Such an increase was found by Schellenberg (2004, 2006). In our study only non-verbal IQ was tested so that we cannot rule out a mediation of crystallized intelligence on the effect of musical training on spelling. However, in this case we would have also expected an effect on reading. The isolated improvement of spelling but not reading suggests a specific link between musical training and spelling abilities mediated by improvement in auditory analysis.

 From our data we propose that there is both an association of musical training and general abilities as well as specific spelling abilities. The link between training and general abilities has been demonstrated to be causal (Schellenberg, 2004). In the retrospective study, Schellenberg (2006) has also shown that the association between duration of musical training and academic average was evident even when IQ was held constant. This, too, suggests a general as well as an additional specific link between musical training and cognitive abilities. Our data justify a prospective study investigating a specific impact of musical training on spelling in languages with shallow orthographies such as German. 
